# The effect of substrate and surface plasmons on symmetry breaking at the substrate interface of the topological insulator Bi_2_Te_3_

**DOI:** 10.1038/s41598-019-42598-9

**Published:** 2019-04-16

**Authors:** Maciej Wiesner, Richard H. Roberts, Jung-Fu. Lin, Deji Akinwande, Thorsten Hesjedal, Liam B. Duffy, Shumin Wang, Yuxin Song, Jacek Jenczyk, Stefan Jurga, Boguslaw Mroz

**Affiliations:** 1 Faculty of Physics, Adam Mickiewicz University, Umultowska 85, PL61614 Poznan, Poland; 2 Microelectronics Research Center, The University of Texas at Austin, TX78757 Austin, USA; 3 Texas Materials Institute, The University of Texas at Austin, 78757 Austin, TX USA; 4 Department of Geological Sciences, Jackson School of Geosciences, The University of Texasat Austin, 78712 Austin, TX USA; 5 Clarendon Laboratory, Department of Physics, Parks Road, University of Oxford, Oxford, OX1 3PU United Kingdom; 6 Key Laboratory of Terahertz Solid State Technology, Shanghai Institute of Microsystem and Information Technology, Chinese Academy of Sciences, Shanghai, 200050 People’s Republic of China; 7 Department of Microtechnology and Nanoscience, Chalmers University of Technology, SE-412 96 Göteborg, Sweden; 8 NanoBioMedical Centre, Adam Mickiewicz University, Umultowska 85, PL 61614 Poznan, Poland

## Abstract

A pressing challenge in engineering devices with topological insulators (TIs) is that electron transport is dominated by the bulk conductance, and so dissipationless surface states account for only a small fraction of the conductance. Enhancing the surface-to-volume ratio is a common method to enhance the relative contribution of such states. In thin films with reduced thickness, the confinement results in symmetry-breaking and is critical for the experimental observation of topologically protected surface states. We employ micro-Raman and tip-enhanced Raman spectroscopy to examine three different mechanisms of symmetry breaking in Bi_2_Te_3_ TI thin films: surface plasmon generation, charge transfer, and application of a periodic strain potential. These mechanisms are facilitated by semiconducting and insulating substrates that modify the electronic and mechanical conditions at the sample surface and alter the long-range interactions between Bi_2_Te_3_ and the substrate. We confirm the symmetry breaking in Bi_2_Te_3_ via the emergence of the Raman-forbidden ${{\boldsymbol{A}}}_{{\bf{1}}{\boldsymbol{u}}}^{{\bf{2}}}$ mode. Our results suggest that topological surface states can exist at the Bi_2_Te_3_/substrate interface, which is in a good agreement with previous theoretical results predicting the tunability of the vertical location of helical surface states in TI/substrate heterostructures.

## Introduction

The Bi_2_Te_3_ family (Bi_2_Te_3_, Bi_2_Se_3_, Sb_2_Te_3_, and Sb_2_Se_3_) of topological insulators (TI) was first predicted by Zhang *et al*.^1^ to have both topologically protected surface states^2^ (TPSS) and an insulating bulk phase^3^, which were later confirmed experimentally. In contrast to the three-dimensional TI Bi_1−x_Sb_x_, which possesses remarkably complex surface states^4^, the surface states of Bi_2_Te_3_ are much simpler, consisting of only a single Dirac cone^5^. This simplicity makes Bi_2_Te_3_ an ideal system for studying the physics of TIs. Moreover, with a band gap of 0.17 eV – well above the room temperature energy – Bi_2_Te_3_ is in principle well-suited for use in electronic devices.

**Figure 1:**
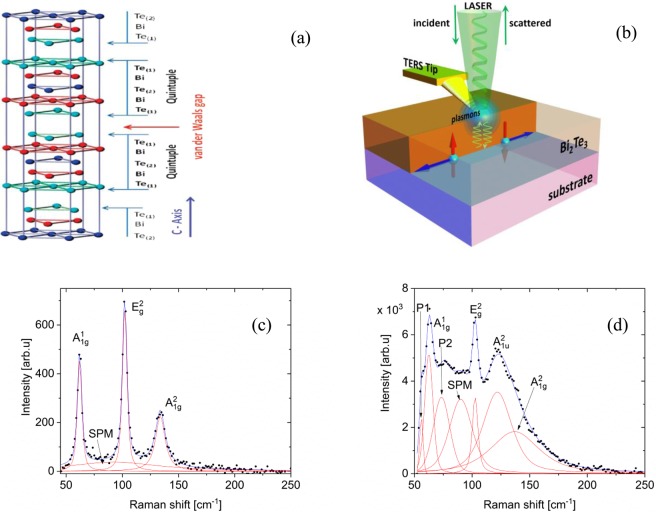
(**a**) Crystal structure of Bi_2_Te_3_ highlighting quintuple layers (QLs) and the van der Waals gap. (**b**) Illustration of the tip-enhanced Raman spectroscopy (TERS) technique. Characteristic (**c**) micro-Raman and (**d**) TERS spectra of Bi_2_Te_3_ on a flat sapphire substrate. The ${A}_{1g}^{1}$ and ${A}_{1g}^{2}$ modes are out-of-plane vibrations with respect to the plane of van der Waals-bonded layers, while the ${E}_{g}^{2}$ mode represents an in-plane vibration. The intensities of the P1 and P2 modes are related to the Bi concentration and the SPM mode to the thickness reduction. The ${A}_{1u}^{2}$ mode is IR-inactive and present in Raman spectra collected from thin TI layers. A detailed description of the modes denoted with red lines is available in the Results section and in the Supplementary Information.

The Bi_2_Te_3_ family also exhibits tunability of thermoelectric properties^6^, phonon dynamics^7^, and charge carrier dynamics by adjusting their thickness^8,9^. Importantly, reducing the thickness in a TI increases the surface-to-volume ratio, which significantly enhances the relative contribution of topological surface states to the measured conductance^5,10^. The crystal structure of the Bi_2_Te_3_ family is characterized by a quintuple layer (QL) structure (Fig. 1a), which is comprised of five atomic, covalently bonded planes, while the QLs are weakly held together van der Waals (vdW) forces. Consequently, Bi_2_Te_3_ can be mechanically exfoliated similarly to graphene, and thicknesses down to a single QL can be achieved^11^. Upon decreasing the thickness to below 80 nm, the loss of infinite crystal periodicity results in the symmetry breaking along the *z*-axis and consequently in the appearance of the Raman-forbidden ${A}_{1u}^{2}$ mode in Raman spectra of exfoliated Bi_2_Te_3_^12,13^.

Bi_2_Te_3_ thin films with broken symmetry have been shown to have topologically non-trivial surface states down to ~3 QLs^14^.

To date, studies have primarily focused on probing topological surface states at the TI/vacuum interface, which requires careful consideration of surface quality and ultra-high vacuum (UHV) conditions^3^. Studies of the TI/substrate interface, however, may relax the technical requirements as the effect of ambient conditions on the interface quality is negligible^15,16^. Still, atomically smooth TI layers grown via molecular beam epitaxy (MBE) are required for investigations of phenomena taking place at the TI/substrate interface. The quality of the interface and charge carrier properties can then be studied using light scattering spectroscopy^17^. Similarly, surface acoustic phonons have been recently employed as a “sonar” probe of electron-phonon coupling at the interface of Bi_2_Te_3_ and GaAs^18^.

Bi_2_Te_3_ has a rhombohedral crystal structure with space group ${D}_{3d}^{5}$ ($R\bar{3}m$) and five atoms in the unit cell. From group theory, Bi_2_Te_3_ has twelve optical branches with the allowed symmetries ${A}_{1g}$, ${A}_{2g}$ ${E}_{g}$, ${A}_{1u}$, ${A}_{2u}$, and ${E}_{u}$. Since Bi_2_Te_3_ is centrosymmetric, the rule of mutual exclusion applies: normal modes cannot be both IR and Raman active^13^. However, IR-active modes in the range of ~50–160 cm^−1^ have been observed in Raman^19,20^ and inelastic He scattering^21^ measurements of Bi_2_Te_3_. These Raman-forbidden and bulk IR modes arise either from breaking of the crystal symmetry in the *z*-direction (due to the limited thickness of a few QLs) or from surface phonons coupling to topological surface states^13^. At present, the symmetry loss in TI thin films is attributed to a large density of domain boundaries formed during coalescence of crystal islands with different lattice orientations, and the Froehlich electron–phonon interaction has been suggested to play a significant role in the Raman scattering processes^21^. However, Li *et al*. showed that symmetry breaking may also result from the fabrication technique. For example, when using the so-called “scotch tape” exfoliation method, the fragmentation of QLs into sub-quintuple layers leads to the emergence of the Raman-forbidden mode A_1u_ in thick slabs of Bi_2_Te_3_^14^.

In this paper, we present a surface-phonon-based micro-Raman and tip-enhanced Raman spectroscopic (TERS) (Fig. 1b) study of interactions between Bi_2_Te_3_ and various substrates on which the TI was grown using MBE. Modifications of the interactions are facilitated by the generation of surface plasmons on various substrates, charge transfer from a semiconducting substrate, and a periodic potential applied to the sample via a corrugated sapphire substrate. These interactions induce symmetry breaking in the *z*-direction of Bi_2_Te_3_, effectively separating the surface properties from the bulk. Symmetry breaking is manifested in the emergence of Raman-forbidden modes, which imply both modified interactions between Bi_2_Te_3_ and substrate and modified long-range interactions between Bi_2_Te_3_ QLs. Our results hint at the possibility of observing topologically protected states at the Bi_2_Te_3_/substrate interface – even for thick Bi_2_Te_3_ samples – which would be a breakthrough for fabrication of nanoelectronics devices for lossless electron transport.

## Results

Micro-Raman measurements of 50-nm-thick Bi_2_Te_3_ grown on a flat, insulating sapphire substrate (Fig. 1c) reveal Raman-active ${A}_{1g}^{1}$, ${A}_{1g}^{2}$, and ${E}_{g}^{2}$ modes in agreement with those previously reported for bulk samples^12^ in the range of ~50–160 cm^−1^. The ${A}_{1g}^{1}$ and ${A}_{1g}^{2}$ modes are out-of-plane vibrations with respect to the plane of van der Waals-bonded layers, while the ${E}_{g}^{2}$ mode represents an in-plane vibration. The ${A}_{1g}$ and ${E}_{g}\,\,$modes can be used to probe the interactions both between and within QLs. It has been shown that with decreasing Bi_2_Te_3_ thickness – and consequent decrease in interlayer interactions – the intensity of the ${A}_{1g}^{2}$ mode increases, reflecting less restrained out-of-plane ${A}_{1g}^{2}$ vibrations^22,23^. This decrease in interlayer interaction for thin films also results in the appearance of a surface phonon mode (SPM)^11^, which is visible in our micro-Raman spectra at 90 cm^−1^.

TERS measurements on the same sample (and substrate) show additional excitations that are absent in micro-Raman spectra (Fig. 1d). Two peaks at ~55 and ~76 cm^−1^ (reported in refs^11,24^), labeled P1 and P2, are observed only in TERS spectra. The most striking difference in the TERS spectra is the emergence of the ${A}_{1u}^{2}$ mode at 119.2 cm^−1^, an IR-active and Raman-forbidden mode that exhibits predominantly out-of-plane atomic motion^25^. Its appearance in Bi_2_Te_3_ Raman spectra has previously been attributed to symmetry breaking in the *z*-direction in sufficiently thin films^11^. The observation of this mode in TERS spectra suggests that LSP generation from the TERS technique can also induce symmetry breaking in Bi_2_Te_3_. LSP generation is evidenced by the more than 10-fold intensity enhancement of TERS spectra compared with micro-Raman spectra^26–28^ (see Fig. 1). Further detail on the Raman peaks observed in micro-Raman and TERS measurements of Bi_2_Te_3_ on sapphire are provided in Table S1.

**Figure 2:**
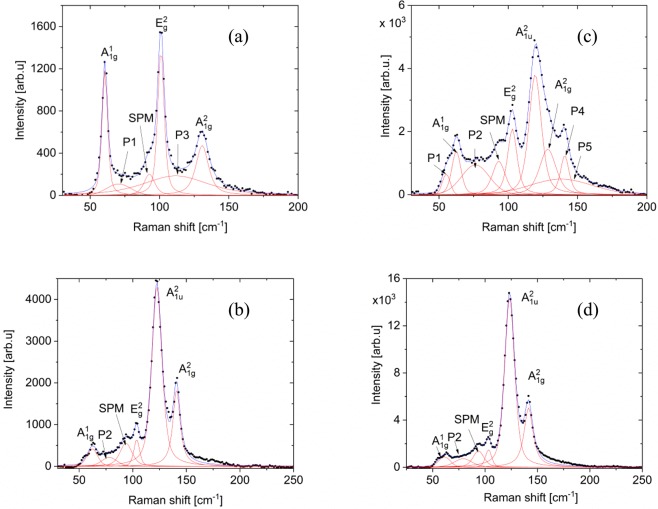
Micro-Raman spectra of Bi_2_Te_3_ on semiconducting (**a**) Si and (**b**) GaAs substrates; TERS spectra of Bi_2_Te_3_ on semiconducting (**c**) Si and (**d**) GaAs substrates. Modes P3 and P4 are related to small stoichiometry variations within the area probed by the laser spot. Mode P5 is usually observed only in Raman spectra of 1–2 QL thick layers^11^.

To confirm that the appearance of the ${A}_{1u}^{2}$ mode is the result of LSP generation and not unique to the sapphire substrate, additional micro-Raman and TERS measurements were performed on 75-nm and 50-nm-thick Bi_2_Te_3_ grown on semiconducting Si and GaAs substrates, respectively. Figure 2a shows micro-Raman spectra for Bi_2_Te_3_ on Si, which features the characteristic ${A}_{1g}^{1}$, ${A}_{1g}^{2}$, and ${E}_{g}^{2}$ modes, as well as a surface phonon mode at ~93 cm^−1^. The unassigned P2 mode is also present in this sample, along with an additional mode at ~108 cm^−1^ (labeled P3). TERS spectra of the same sample (Fig. 2c) show the emergence of the Raman-forbidden ${A}_{1u}^{2}$ mode, providing further evidence that LSP generation from TERS induces symmetry breaking.

A large signal from the characteristic mode of the Si substrate at ~520 cm^−1^ is observed via TERS measurements, but not in micro-Raman spectra (Fig. S1). Observation of the Si mode suggests that the measurement is quite sensitive to the Bi_2_Te_3_/Si substrate interface – a result of the difference in the penetration depth for Raman and TERS measurements. The light penetration depth is given by $l=\sqrt{\pi fn{\mu }_{e}{\mu }_{m}}\,$, where *f* is light frequency, ${\mu }_{e}$is the electron mobility, ${\mu }_{m}$is the magnetic permeability, and *n* is the electron concentration. We suggest that TERS-induced localized surface plasmons increase the local electron concentration *n*, leading to an increase of the light penetration depth and consequent appearance of the Si peak.

Micro-Raman spectra of 50-nm-thick Bi_2_Te_3_ grown on GaAs also exhibit the characteristic Raman-active modes, SPM, and unassigned P2 mode (Fig. 2b). Unlike samples grown on sapphire and Si substrates, however, the Raman-forbidden ${A}_{1u}^{2}$ mode is visible *without* TERS-induced plasmon generation. Consequently, another mechanism must be responsible for symmetry breaking in this sample. As will be further discussed below, a plausible mechanism is charge transfer from the GaAs substrate. TERS spectra on the same sample are nearly identical, but with larger Raman intensities due to the characteristic signal enhancement of the technique (Fig. 2d). Further details of the fittings of Raman spectra for Bi_2_Te_3_ on Si and GaAs are given in Tables S2 and S3, respectively.

**Figure 3:**
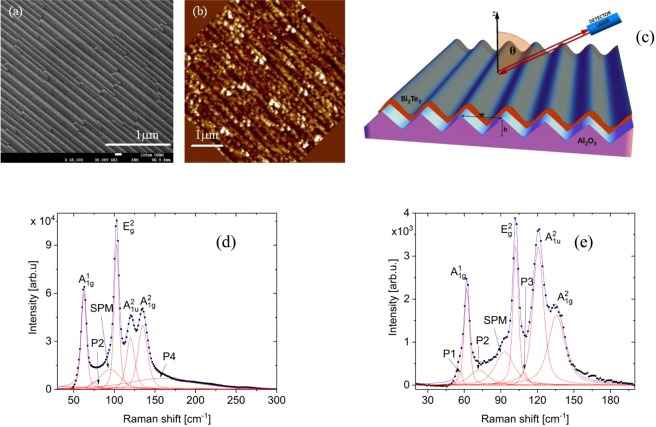
(**a**) Scanning electron micrograph of a sapphire surface with corrugation period *w* = 250 nm and height *h* = 20 nm. (**b**) Atomic force micrograph of a 30-nm-thick Bi_2_Te_3_ film on the corrugated sapphire substrate. (**c**) Schematic of the experimental geometry for micro-Raman measurements of Bi_2_Te_3_ grown on a corrugated sapphire substrate with *w* = 250 nm, *h* = 20 nm, and *θ* = 10°. (**d**) Micro-Raman and (**e**) TERS spectra for a 30-nm-thick Bi_2_Te_3_ film on corrugated sapphire with the geometry shown in (**c**).

Finally, we examine the effect of a periodic strain potential on 30-nm-thick Bi_2_Te_3_ films grown on a corrugated sapphire substrate. Due to the instability of sapphire’s *m*-plane surface when annealed at high temperatures, it undergoes spontaneous faceting that results in the formation of V-shaped nanogrooves. Our annealing procedure resulted in substrates with corrugation height and period of *h* = 20 nm and *w* = 250 nm, respectively (Fig. 3a). Bi_2_Te_3_ was then grown directly onto the corrugated substrate to induce a periodic strain potential. Further details of the procedure are provided in Supporting Information.

Micro-Raman measurements of Bi_2_Te_3_ grown on corrugated sapphire were carried out with the laser at an incident angle of *θ* = 10° with respect to the plane of the substrate (see Fig. 3c). This was done to maintain the same scattering geometry as micro-Raman measurements on flat substrates, compensating for the corrugation angle. For TERS measurements, it is more important that the laser beam is properly focused on the apex of the metallic tip shown in Fig. 1b, rather than on the sample surface, and so the standard angle of *θ* = 0° was used. Both micro-Raman and TERS spectra reveal ${A}_{1u}^{2}$ mode emergence (Fig. 3d,e), suggesting that symmetry breaking is induced by the applied periodic strain potential. Additional details of the modes fitted in Fig. 3d,e are listed in Table S4.

## Discussion

Broken symmetry of a TI can lead to separation of bulk and surface conduction, and exploiting this phenomenon provides a crucial step forward for experimental studies of TIs and realization of TI-based devices. However, the design process of nanoelectronics devices based on TIs should take into consideration thermodynamic conditions for which the devices are designed. It is a well-known fact that at elevated temperatures, quantum effects are washed out, and, consequently the special properties of the electrically conducting surfaces disappear. Therefore, to suppress the thermal excitation of charge carriers from the bulk into TIs surface states, the energy gap of the system must be increased.

An excellent candidate for nanoelectronic devices is Bi_2_Se_3_ with a band gap of 0.3 eV, which is twice the value of Bi_2_Te_3_ of 0.15 eV^1^, which makes the observation of TI behavior at room temperature more robust. On the other hand, by decreasing the thickness of Bi_2_Te_3_ down to 1 QL, its energy gap can reach 0.45 eV^14,29^. This implies suppression of contribution of the bulk electrons into surface states of thin TI layers. Moreover, thermal excitations are related to scattering by acoustic phonons (AP) and decreasing the TI thickness leads to a reduction of bulk AP. Finally, for sufficiently thin TI layers (between 3 QL’s and 9 QL’s), topologically protected surface states appear^30^ and only surface phonons remain. The electron-surface AP coupling^18^ can lead to spin-like oscillations of electrons, which can be exploited in device applications. Here, we demonstrated that not only thickness reduction leads to the symmetry breaking, but also LSP generation and interactions of the TI with different substrates. Therefore, we expect that Bi_2_Te_3_– based devices, grown on selected substrates, will preserve the unique transport properties at the TI/substrate interface even at room temperature. Such conducting edge states at room temperature were recently reported in the two-dimensional TI bismuthene grown on SiC^31^.

Previous work has demonstrated that reducing the thickness of Bi_2_Te_3_ results in symmetry breaking along he *z*-axis of the material, and consequently the emergence of the Raman-forbidden ${A}_{1u}^{2}$ mode and SPM^11–13,24,32^. Due to the broken symmetry, various surface phenomena have been observed in ultrathin (1-2 nm) Bi_2_Te_3_^9,12^. Symmetry-breaking in Bi_2_Te_3_ can also be induced by interactions with the substrate: the effect of a magnetic substrate on symmetry breaking was presented in ref.^33^, in which the emergence of ferromagnetism in the bottom surface of Bi_2_Se_3_ was demonstrated by observation of an additional Shubnikov–de Haas frequency.

In our experiments, we demonstrate three additional mechanisms for symmetry breaking: surface plasmon generation, charge transfer, and the presence of a periodic potential. By employing micro-Raman and TERS spectroscopy, we link the emergence of Raman-forbidden optical phonon modes to underlying broken symmetry of Bi_2_Te_3_.

It is well established that the TERS technique uses the local plasmon mode of a sharp metallic nanotip to confine and enhance the electric field near the tip apex. LSP generation in the sample occurs via tip-sample coupling and tip-induced sample heating, which can elevate the sample’s temperature^34–36^. The excellent thermoelectric properties of Bi_2_Te_3_ result in localization of thermally activated electric charges. This, paired with the photoelectric effect from the incident laser light^37–40^, results in an increase in local charge density and increases the light penetration depth. We attribute the appearance of the characteristic Raman mode of the Si substrate to this effect, and it is clear evidence of electron-phonon coupling at the Bi_2_Te_3_/Si interface. A similar effect has been observed with Bi_2_Te_3_ on a ZnO substrate, for which surface plasmons enhance the photoluminescence from ZnO^41^.

Surface plasmons in Bi_2_Te_3_ can also be generated due to various inter- and intra-band transitions, including bulk interband transitions in the visible range, intraband transitions within topologically protected surface bands in the mid-infrared, and interband transitions between bulk states and topologically protected surface states spanning the UV to near-infrared^35^. The existence of such surface plasmons in Bi_2_Te_3_ has been confirmed by High Resolution Transmission Microscopy (HRTEM) measurements^28^. In our experiments, LSP generation is evidenced by roughly three- and ten-fold intensity enhancements in TERS measurements compared with micro-Raman for Bi_2_Te_3_/Si and Bi_2_Te_3_/flat sapphire samples, respectively. Additionally, LSP generation results in a symmetry breaking of the material evidenced by the appearance of the Raman-forbidden ${A}_{1u}^{2}\,\,$mode in TERS spectra.

We also show that electron transfer from the substrate can break the symmetry of Bi_2_Te_3_, as we observe of the ${A}_{1u}^{2}$ mode in both micro-Raman and TERS measurements on a GaAs substrate (Fig. 2). The work functions for GaAs and Bi_2_Te_3_ are 4.69 eV and 5.3 eV, respectively. Thus, once the materials are in contact, electrons transfer from GaAs to Bi_2_Te_3_, leading to a large increase in electron density^18,42,43^. The charge transfer from a substrate to a TIis also responsible for tuning vertical location of helical surface states^23^. Wu *et al*. assert that the substantial electronic hybridization at the interface decreases coupling between the first and second QL of the TI, shifting the topologically protected states upward from the first to the second QL.

The ${A}_{1u}^{2}\,\,$mode was also observed in both micro-Raman and TERS spectra collected from Bi_2_Te_3_ on a corrugated sapphire substrate (Fig. 3e). We suggest that the substrate corrugation induces sufficient strain in Bi_2_Te_3_ to result in symmetry breaking^44^. This agrees with ref.^45^, which reports that tensile and compressive deformations of Bi_2_Te_3_ QLs can cause a shift in the atomic layers of Bi and Te and, as a result, a reduction in symmetry. More detailed theoretical investigations have shown that the lattice constant of Bi_2_Te_3_ increases at a rate of 0.012 Å per 1% of in-plane uniaxial strain ranging between −6% to 6% (compressive to tensile)^46^ and the band gap increased from 0.07 to 0.16 eV between −3 to 3% strain^43^. Strain also induces flexoelectricity and subsequent electric polarization in Bi_2_Te_3_ – a signature of symmetry breaking in the z-direction^47^. Therefore, uniaxial strain induced by the corrugated substrate can alter the properties of Bi_2_Te_3_ through both symmetry breaking and strain-induced modifications to the band structure.

Optical phonons are a common tool for probing intra- and inter-layer interactions between van der Waals-bonded layers such as those in Bi_2_Te_3_ QLs. Previous studies have demonstrated that the ${A}_{1g}^{1}$ and ${A}_{1g}^{2}\,\,$modes redshift and blueshift, respectively, with decreasing Bi_2_Te_3_ thickness^11,48^ and the intensity ratio $I=\frac{I({A}_{1g}^{2})}{I({E}_{g}^{2})}$ increases with decreasing thickness due to less restrained out-of-plane ${A}_{1g}^{2}\,\,$vibrations^13^. This indicates that the long-range interaction between QLs is weakened as the thickness decreases. The SPM is also a sensitive indicator of Bi_2_Te_3_ thickness, as it has been shown that the mode increases in intensity as thickness is reduced from 40 nm to a single QL. We observed the SPM mode in all collected micro-Raman and TERS spectra, providing further evidence of weak interactions between QLs in investigated samples. Furthermore, since the out-of-plane and surface modes in Bi_2_Te_3_ are sensitive to the interaction between QLs, one can use them to derive information about symmetry breaking in the direction perpendicular to the QLs.

Analysis of the intensity ratio *I* of Bi_2_Te_3_ on various substrates revealed that local surface plasmon generation, charge transfer, and a periodic strain potential all act to increase *I* (Table 1). This implies that these mechanisms decrease interlayer interactions in the material – a phenomenon that was previously only associated with thickness reduction in Bi_2_Te_3_. In each case, the ratio increase is primarily due to an increase in ${A}_{1g}^{2}$ mode intensity rather than a decrease in ${E}_{g}^{2}$ mode intensity, suggesting that out-of-plane ${A}_{1g}^{2}$ vibrations become less restrained due to weaker interlayer bonding. This effect is most pronounced for the charge transfer mechanism, as *I* for Bi_2_Te_3_ on GaAs is nearly an order of magnitude larger than for Si or flat sapphire substrates. For LSP generation and strain mechanisms, the effect is more modest: values of *I* based on TERS measurements were ~30% larger than those for micro-Raman and ~10% larger for measurements on corrugated compared with flat sapphire. Additionally, the ${A}_{1g}^{2}$ mode was found to blueshift by an average of ~3.5 cm^−1^ in TERS measurements compared with micro-Raman (Tables S1-S4), as would be expected for Bi_2_Te_3_ exhibiting weaker interlayer bonding.

**Table 1:** Ratio of $I({A}_{1g}^{2})/I({E}_{g}^{2})$ for samples investigated with micro-Raman and TERS.

Substrate	${\boldsymbol{I}}({{\boldsymbol{A}}}_{{\bf{1}}{\boldsymbol{g}}}^{{\bf{2}}})/{\boldsymbol{I}}({{\boldsymbol{E}}}_{{\boldsymbol{g}}}^{{\bf{2}}})$	
	micro-Raman	TERS
Si	0.40	0.84
GaAs	2.86	3.39
Sapphire corrugated	0.38	0.49
Sapphire flat	0.34	0.55

Based on the emergence of the Raman-forbidden ${A}_{1u}^{2}$ mode and changes in intensities and frequencies of Raman-active optical modes, one can conclude that LSP generation, charge transfer, and application of a periodic potential can each modify the interactions between individual QLs and break the symmetry of bulk Bi_2_Te_3_. Such effects – which have previously only been observed in Bi_2_Te_3_ thin films – suggest that isolation of surface phenomena is achievable in bulk Bi_2_Te_3_ via proper selection of substrate and experimental technique.

## Conclusions

The analysis presented herein has shown that LSP generation, charge transfer, and application of a periodic potential can modify the long-range interactions between QLs in a Bi_2_Te_3_ sample near the substrate interface. This leads to the emergence of Raman-forbidden modes and enhanced out-of-plane vibrations characteristic of topologically insulating Bi_2_Te_3_ thin films with broken symmetry. Our results highlight the need for further investigations of the quantum Hall effect in Bi_2_Te_3_ samples with broken symmetry and raise the possibility of isolating topologically protected surface states from bulk states at the interface between Bi_2_Te_3_ and a substrate – a potential breakthrough for engineering lossless devices based on TIs.

The periodic strain introduced by corrugation causes density fluctuations of the TI layer leading to transverse spin fluctuation^49,50^. For thin TI layers, charge-like and spin-like plasmons can be distinguished, as the first couple to optical and the latter to acoustic phonons, respectively^50^. Investigations of the acoustic phonon dispersion in TIs with and without magnetic field should be able to validate the spin-charge separation hypothesis.

Next, electron transport measurements addressing the Bi_2_Te_3_/substrate interface should be undertaken to determine if surface conduction can be isolated from bulk via the mechanisms discussed in this work.

## Methods

### Sample fabrication

Bi_2_Te_3_ thin films were grown via MBE on Si(111), GaAs (001) with a 2° offcut towards [110], and *m*-plane $[101\bar{0}]$ cut sapphire (α-Al_2_O_3_) substrates with flat and corrugated surfaces. The growth temperature was kept at 220 °C. The thicknesses of the Bi_2_Te_3_ films grown on these substrates were as follows: 75 nm on Si, 50 nm on GaAs, and 30 nm and 50 nm on sapphire substrates. Corrugated sapphire substrates were fabricated using a special heat-treatment procedure that results in surface reconstruction^51–53^. Further details of the sample fabrication are presented in the Supporting Information.

### Tip-Enhanced Raman Spectroscopy (TERS) and micro-Raman Spectroscopy

For TERS measurements, a Renishaw inVia spectrometer and NTMDT TERS system were employed in the top-illumination and top-collection type geometry and equipped with a 3 mW, 633-nm wavelength laser.

Micro-Raman spectra were measured in a backscattering configuration using a commercial Renishaw inVia micro-Raman system and a 3 mW, 633 nm wavelength laser. All spectra were measured under 50x magnification resulting in a beam spot about 0.7 μm in diameter. A spectral resolution of about 1 cm^−1^ was achieved using a 1200 l/mm grating. Additional micro-Raman spectra were collected using the NTMD system with a Renishaw spectrometer to compare with TERS measurements; spectra were collected in the tip-retracted position to acquire only the far-field Raman component. The Raman spectra collected using these two systems were comparable.

Details on the TERS technique can be found in the Supporting Information.

## Supplementary information

The effect of substrate and surface plasmons on symmetry breaking at the substrate interface of the topological insulator Bi2Te3

## Data Availability

Data from Raman and TERS measurements are available upon request to M.Wiesner, mwiesner@amu.edu.pl.
