# Supplementary material for: The effect of substrate and surface plasmons on symmetry breaking at the substrate interface of the topological insulator Bi2Te3
Source: Sci Rep. 2019 Apr 16;9:6147. doi: 10.1038/s41598-019-42598-9 (PMC6468116; doi:10.1038/s41598-019-42598-9)
Supplement: Supplementary file 1 — The effect of substrate and surface plasmons on symmetry breaking at the substrate interface of the topological insulator Bi2Te3 [file 41598_2019_42598_MOESM1_ESM.pdf]

Supporting Information

The effect of substrate and surface plasmons on symmetry breaking at the substrate interface of the topological insulator Bi_2Te_3

Maciej Wiesner[1,2], Richard H. Roberts[2,3], Jung-Fu. Lin[3,4], Deji Akinwande[2,3], Thorsten Hesjedal[5], Liam B. Duffy[5], Shumin Wang[6,7], Yuxin Song[6], Jacek Jenczyk[8], Stefan Jurga[8], Boguslaw Mroz[1]*

[1] Faculty of Physics, Adam Mickiewicz University, Umultowska 85, PL61614, Poznan, Poland

[2] Microelectronics Research Center, The University of Texas at Austin, Austin, TX 78757, USA

[3] Texas Materials Institute, The University of Texas at Austin, Austin, TX 78757, USA

[4] Department of Geological Sciences, Jackson School of Geosciences, The University of Texas at Austin, Austin, TX 78712, USA

[5] Clarendon Laboratory, Department of Physics, Parks Road, University of Oxford, Oxford OX1

3PU, United Kingdom

[6] Key Laboratory of Terahertz Solid State Technology, Shanghai Institute of Microsystem and Information Technology, Chinese Academy of Sciences, Shanghai 200050, People's Republic of China

[7] Department of Microtechnology and Nanoscience, Chalmers University of Technology, SE-412 96, Göteborg, Sweden

[8] NanoBioMedical Centre, Adam Mickiewicz University, Umultowska 85, PL 61614 Poznan, Poland

*Correspondence should be addressed to mwiesner@amu.edu.pl

S1. Substrate selection

Two semiconducting substrates, Si and GaAs, and both flat and corrugated insulating sapphire substrates were used to investigate substrate effects on the optical phonon dynamics of topological insulator Bi_2Te_3. Since the work functions of Si and GaAs are smaller than the one for Bi_2Te_3, charge transfer from the substrate to the sample can affect the band structure of Bi_2Te_3 and, as a result, affect the electron-phonon coupling[12,18,35,47]. Insulating sapphire substrates were used to suppress the electron transfer from the substrate, and corrugated sapphire substrates were used to induce a periodic strain potential in Bi_2Te_3[S1,S2]. Such periodic structures can be effective phononic materials: with the proper choice of material parameters and modulation

spacing, it is possible to induce a complete phononic band gap for which propagation of acoustic waves of arbitrary polarization and wave vector is forbidden. Furthermore, two-dimensional periodic nanostructures enable the control of heat flow and propagation of hypersonic surface acoustic waves[S3].

Periodic structures can also lead to anisotropic electron transport and altered band structures (e.g., in graphene[S4-S5]) and energy gap opening[S6]. The periodic potential induced by such structures is also a source of strain. This has been demonstrated for thin layers of MoS_2 transferred onto an array of artificial nanostructures and by deposition of a nanostructure on top of the 2D material[S7- S12]. It is well known that sample/substrate lattice-mismatch can be a source of strain for epitaxially grown samples. In our case, the mismatch between the corrugated sapphire substrate and the Bi_2Te_3 layer results in sufficient strain to break the symmetry of the TI in the *z*-direction. Thus, significant differences between Raman spectra collected from the flat and the corrugated sapphire substrates were observed. Corrugation on the sapphire surface can be achieved due to the instability of the *M*-plane of sapphire (α-Al_2O_3) when annealed at high temperatures. Under such conditions, sapphire undergoes a spontaneous faceting, resulting in nanogroove formation[52-54]. The saw-tooth-shaped structures have a triangular base width of 250 nm and a height of 20 nm. To achieve this groove geometry, we annealed the sapphire at 1500 ^{o}C for 22 hours.

S2. Bi_2Te_3 growth

Bi_2Te_3 thin films were grown by MBE on Si(111), GaAs(001) with a 2° off-cut towards [111], and $(10\bar{1}0)$ *m*-plane off-cut sapphire (α-Al_2O_3) with both flat and a corrugated surfaces. The Si substrate was etched by a mixed chemical solution of HF and NH_2F to remove most of the

surface oxide, then quickly loaded into the MBE growth chamber[S13]. The substrates were subsequently annealed at elevated temperatures (600°C for Si, 650°C for GaAs, and 700°C for sapphire) to remove the surface oxide. To passivate the dangling bonds of the semiconductor substrates prior to film growth, substrates were exposed to Te for 1 min at elevated temperature. Bi_2Te_3 growth temperatures were 180°C for the GaAs substrate and 220°C for Si and sapphire substrates. The beam equivalent pressures (BEP) of Te and Bi were 10^{-6} Torr and 10^{-8} Torr, respectively, resulting in a growth rate of 50 nm/h. For growth on Si, first a seed layer was grown at a lower temperature of 180°C for 4 min before the temperature was ramped up to 220°C at a rate of 15°C/min. The total thickness was 75 nm. For the growth on GaAs, no seed layer was used. Instead, the "quintuple layer (QL) by QL" method was employed, whereby the film is grown in increments of 0.5 QL, followed by 30 s of Te soaking. This sequence was repeated 100 times, resulting in total film thickness of 50 nm. For the growth on sapphire, the growth temperature was kept at 220°C [S14]. The thicknesses of the Bi_2Te_3 films were 30 nm and 50 nm on flat and corrugated sapphire substrates, respectively.

S3. Experiment

A Renishaw inVia micro-Raman spectrometer combined with a NTMDT system were employed for micro-Raman and tip-enhanced Raman spectroscopy (TERS) measurements, respectively. Micro-Raman studies were made using laser wavelengths of 633 nm and 473 nm, whereas TERS measurements were made with a 633-nm wavelength laser only. The laser power in both experiments was 3 mW. To compare the measurement results obtained with both techniques in a meaningful way, here we only present experimental data collected with a 633-nm wavelength laser.

For the experiments on Bi_2Te_3 on corrugated sapphire the laser beam polarization was perpendicular to the corrugation direction.

The TERS technique – which combines atomic force microscopy (AFM), Raman light scattering, and near-field microscopy – is used for investigations of Raman scattering at the nanoscale, far beyond the diffraction limit of the probing light. In TERS, a metallic tip (usually silver or gold) with a tip radius of 10-20 nm is brought into feedback with the surface of a sample using a typical atomic force or scanning tunneling microscope setup. We used a top-illumination and top-collection type of TERS technique. The TERS technique is enabled by the excitation of localized surface plasmons that are confined at the sharp apex of a metallic tip located inside the focal region of a tightly focused laser beam. The technique utilizes the local mode of surface plasmons of the nanotip to confine and enhance the light field near the tip apex, which can be used to excite Raman scattering from a nanoscopic volume of a sample placed under the tip. The Raman scattering process takes place in the near-field and the spectral signal is scattered and converted back to the far-field by the tip apex, which is then collected by standard optics and a spectrometer in the far-field.

The short distance between the TERS tip and the sample surface can result in near-field tip-sample coupling, which allows for surface plasmon excitation in the sample[S15]. We use this phenomenon to explain the generation of localized surface plasmons (LSPs) in our samples and subsequent emergence of Raman-forbidden modes in thick Bi_2Te_3 layers. Excitation of LSPs near the tip results in the creation of a field-source at the tip apex that produces a localized, evanescent field. This field, in addition to the incident laser beam focused a few nanometers over the sample surface, results in sample heating[35-37]. These two effects significantly increase the sample's temperature and, as a result – considering the excellent thermoelectric properties of the Bi_2Te_3 –

one can expect a dramatic increase in the density of thermally activated electric charges at the vicinity of the tip apex. We propose that this effect induces symmetry breaking along *z*-direction of the sample.

Additional mechanisms for LSP generation in the sample surface include: bulk interband transitions contributing primarily in the visible spectral region, intraband transition within topologically protected surface bands contributing in the mid-infrared region, and interband transition between topologically protected surface states and bulk states that dominate in the UV–near-infrared range[28].

The existence of LSPs in Bi_2Te_3 have previously been confirmed by HRTEM measurements[24], and the effect of LSPs on the enhancement of photoluminescence of ZnO has also been reported[42]. From results presented in the main text, we are unable to deduce which mechanism of LSP generation dominates in our experiments. Nevertheless, the effect of LSP generation is evident in the signal enhancement apparent in TERS spectra compared with micro-Raman experiments.

Since the laser spot of the micro-Raman system is larger than the TERS tip apex, each signal in the TERS measurement is composed of a tip-enhanced component (near-field) and an unenhanced out-of-tip component (far-field). The micro-Raman spectra were collected in the tip-retracted position to acquire only the far-field Raman component. For comparison, the micro-Raman spectra were also collected using an inVia micro-Raman system and an objective with 50x magnification. Collected spectra from each system were comparable.

S4. Results

Measured positions of the vibrational modes observed in micro-Raman and TERS spectra (Figure S1) are presented in Tables S1-S4. Unassigned modes P1-P5 have previously been described in Refs. 14 and S16.

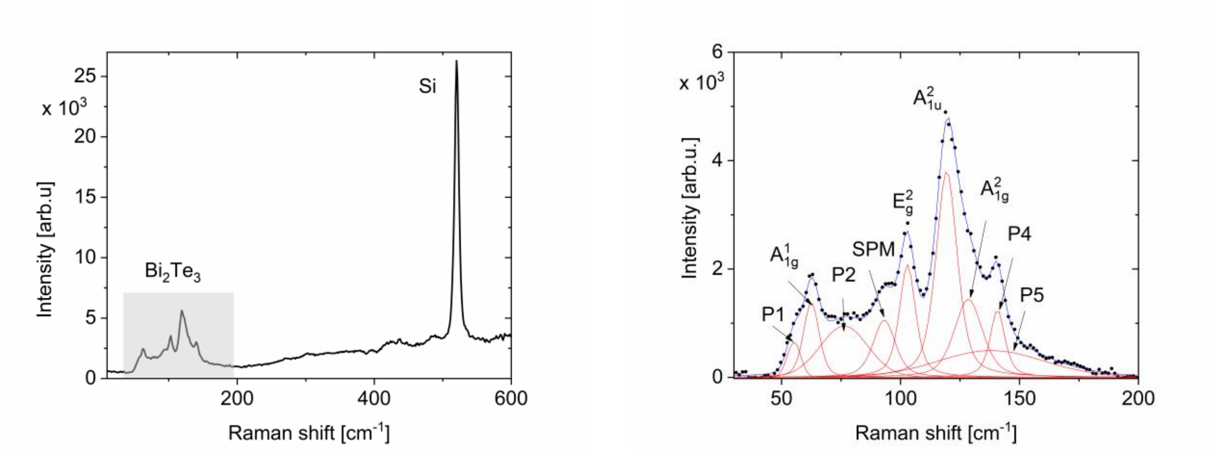

Figure S1. (a) Results of TERS measurements on a Bi_2Te_3/Si sample (t = 75 nm) and (b) peak fitting of the area marked in grey in (a).

Table S1. Assignment of Raman peaks observed in micro-Raman and TERS measurements of Bi_2Te_3 film of h=50nm grown on the flat sapphire substrate (see Figure 1). Units: cm^{-1}.

Vibration	P1	A_{1g}^1	P2	SPM	E_g^2	A_{1u}^2	A_{1g}^2
micro-Raman	-	62.0	-	92.6	102.1	-	133.8
TERS	55.9	62.4	73.5	90.1	102.6	121.9	136.9

Table S2. Assignment of Raman peaks observed in micro-Raman and TERS measurements of Bi_2Te_3 film of h=75 nm grown on the Si substrate (see Figure 2a and 2c). Units: cm^{-1}.

Vibration	P1	A_{1g}^1	P_2	SPM	E_g^2	P3	A_{1u}^2	A_{1g}^2	P4	P5
micro-Raman	-	60.5	71.1	94.6	101.0	107.8	-	130.4	-	
TERS	55.2	62.5	76.6	93.2	102.9	-	119.7	128.6	140.7	141.6

Table S3. Assignment of Raman peaks observed in micro-Raman measurements of Bi_2Te_3 film of h=50nm grown on the GaAs substrate (see Figure 2b and 2d). Units: cm^{-1}.

Vibration	A_{1g}^1	P_2	SPM	E_g^2	A_{1u}^2	A_{1g}^2
micro-Raman	62.3	78.0	93.6	103.7	122.5	140.9
TERS	61.2	79.1	93.7	103.3	123.4	141.5

Table S4. Assignment of Raman peaks observed in TERS measurements of Bi_2Te_3 film of h=30nm grown on the corrugated sapphire substrate (see Figure 3d and 3e). Units: cm^{-1}.

Vibration	P1	A_{1g}^1	P_2	SPM	E_g^2	P3	A_{1u}^2	A_{1g}^2
micro-Raman	-	62.5	75	94	102.8	-	120	135.1
TERS	56.4	62.0	72.0	93.1	102.2	108.9	120.7	135.8

Supporting References

S1. Graczykowski, B.;, Mielcarek, S.; Trzaskowska, A.; Sarkar, J.; Hakonen, P.; Mroz, B. 2016, Tuning of a hypersonic surface phononic band gap using a nanoscale two-dimensional lattice of pillars, *Phys. Rev. B*, **86**, 085426.

S2. Trzaskowska A.; Mielcarek, S.; Wiesner, M. 2014, One-dimensional modulation of the stripe in a surface phononic lattice: The effect on propagation of surface waves, *J. of Appl. Phys*., **116**, 214303.

S3. Shaner, E. A.; Leseman, Z. C.; Serrano, J. R.; Phinney, L. M.;, El-Kady, I. 2011, Reduction in the Thermal Conductivity of Single Crystalline Silicon by Phononic Crystal Patterning, *Nano Lett.,* **11***,* 107.

S4. Park, Ch.; Yang, L.; Son, Y.; Cohen, M. L.; Loulie, S. G. 2008, Anisotropic behaviours of massless Dirac fermions in graphene under periodic potentials, *Nat. Phys*., **4** ,213.

S5. Park, Ch.; Tan, L. Z.; Louie, S. G. 2011, Theory of the electronic and transport properties of graphene under a periodic electromagnetic field, *Physica E*, **43**, 651.

S6. Xie, W.; Li, Z. 2016, Energy band gaps in periodic bent graphene, *Solid State Communications*, **225**, 22.

S7. Shin, B. G.; Han, G. H.; Joon Yun, S. Min, H., *et al.* 2016, Indirect Bandgap Puddles in Monolayer MoS_2 by Substrate-Induced Local Strain *Bong Adv. Mater.*, **28**, 9378.

S8. Li, H.; Contryman, A. W.; Qian, X.; Moeini Ardakani, S.; Gong, Y.; Wang, X. *et al.* 2015, Optoelectronic crystal of artificial atoms in strain-textured molybdenum disulphide *Nat. Comm.*, **6**, 7381.

S9. Castellanos-Gomez, A.; Roldán, R.; Cappelluti, E.; Buscema, M.; Guinea, F., van der Zant, H. S. J.; Steele, G. A. 2013, Local Strain Engineering in Atomically Thin MoS_2, *Nano Lett*., **13**, 5361.

S10. Saradhi Mangu, V.; Zamiri, M.; Brueck, S. R. J.; Cavallo, F. 2017, Strain engineering, efficient excitonic photoluminescence, and exciton funnelling in unmodified MoS_2 nanosheets *Nanoscale*, **9**, 16602.

S11. Feng, J.; Qian, X.; Huang, Ch.; Li, J. 2012, Strain-engineered artificial atom as a broad-spectrum solar energy funnel, *Nat. Phot*., **6**, 866.

S12. Moghadasi, A.; Roknabadi, M. R.; Ghorbani, S. R.; Modarresi, M. 2017, Electronic and phononic modulation of MoS_2 under biaxial strain, *Physica B*., **526**, 96

S13. Fülöp, A.; Song, Y.; Charpentier, S.; *et al.* 2014, *Appl. Phys. Express*, **7**, 45503.

S14. Virwani, K.; Harrison, S. E.; Pushp, A. *et al.* 2014, Controlled removal of amorphous Se capping layer from a topological insulator, *Appl. Phys. Lett.*, **105**, 241605.

S15. Behr, N.; Raschke, M. B. 2008, Optical Antenna Properties of Scanning Probe Tips: Plasmonic Light Scattering, Tip-Sample Coupling, and Near-Field Enhancement, *J.Phys.Chem.C,* **112**, 3766.

S16. Rui, H.; Sukrit, S.; Zhipeng, Y.; Courtney, K.; Kidd, T. E.; Xuan, P.; Gao, A. 2015, Laser induced oxidation and optical properties of stoichiometric and non-stoichiometric Bi_2Te_3 nanoplates, *Nano Research*, **8**, 851.
